# Microvascular Damage in a Young Female Archer Assessed by Nailfold Videocapillaroscopy: A Case Report

**DOI:** 10.3390/ijerph17124218

**Published:** 2020-06-12

**Authors:** Maria Maddalena Sirufo, Enrica Maria Bassino, Francesca De Pietro, Lia Ginaldi, Massimo De Martinis

**Affiliations:** 1 Department of Life, Health and Environmental Sciences, University of L’Aquila, 67100 L’Aquila, Italy; maddalena.sirufo@gmail.com (M.M.S.); enricamaria.bassino@gmail.com (E.M.B.); fra722@hotmail.it (F.D.P.); lia.ginaldi@cc.univaq.it (L.G.); 2 Allergy and Clinical Immunology Unit, Center for the diagnosis and treatment of Osteoporosis, AUSL 04 Teramo, 64100 Teramo, Italy

**Keywords:** archery, woman’s health, microcirculation, nailfold capillaroscopy, autoimmune disease, sport injuries, microvascular damage

## Abstract

Archers are known to be exposed to the risk of developing various injuries, including less described microvascular damages, which can however heavily affect the performance of athletes. Nailfold videocapillaroscopy is a safe, proven and non-invasive method that allows us to examine the nail capillaries and diagnose vascular anomalies in athletes suffering from the consequences of microtrauma caused by repeated use of fingertips. The detection of defined capillaroscopic pictures is the basis for the follow-up and suggests carrying out further clinical investigations to exclude underlying pathologies. In women this aspect is even more important since they are more frequently affected by autoimmune diseases such as scleroderma which can cause microcirculation alterations. We report the case of a 16-year-old woman who has been practicing archery for five years. She had been complaining for two years about painful fingertips, worsening in the last year. Through videocapillaroscopy, carried out by using a ×200 optical probe-equipped videocapillaroscope connected to image analyzer software (VideoCap software 3.0; DS Medica, Milan, Italy), we detected changes in the microvasculature compatible with a non-specific pattern. The findings of these anomalies suggest a diagnostic analysis aimed at excluding the presence of systemic diseases such as scleroderma. Once these conditions are excluded, and assuming that the documented alterations are due to the particular muscular effort and vibrations to which the fingers are subjected in shooting, we suggest follow-up to keep under control possible further developments and clinical changes. As far as we know, this is the first report that documents and describes the condition of microvascular changes in an archer. Archers, similar to other athletes who mainly use fingertips such as volleyball players, are more exposed to the development of digital traumas that can induce alterations in the microcirculation. We suggest that a periodic capillaroscopy should be included in the health surveillance program of these athletes, in fact this simple, reliable, non-invasive and inexpensive diagnostic tool is able to recognize early signs of microvascular damage and then suggest indications for further investigations and or follow-up.

## 1. Introduction

Evidence of ancient archers has been found around the world [1,2] and archery is one of the oldest arts still practiced today. The evolution of archery began at the start of mankind’s history and was also documented in a famous drawing by Leonardo da Vinci. First introduced as an Olympic sport at the Games of the II Olympiad in Paris in 1900, subsequently excluded and then reinserted in 1972 at the Monaco Olympic Games [3,4]. It is reported that in 2017 the number of participants in archery in the United States amounted to approximately 7.77 million [5].

**Figure 1:**
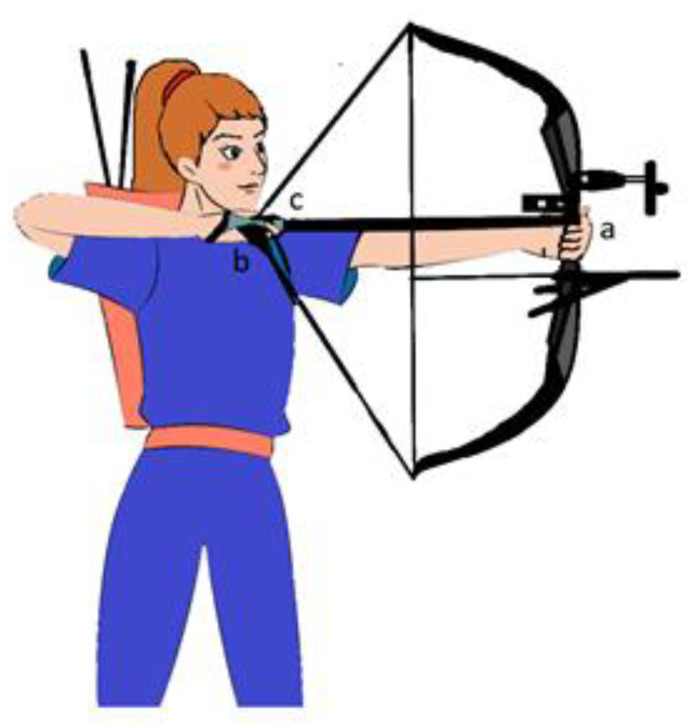
Parts of the arch compound and shooting technique: (**a**) riser, (**b**) rope and (**c**) arrow. The hooking of the back of the arrow (**c**) to the rope (**b**) it is done by placing first the third finger and fourth finger of the right hand and then the second finger of the same hand. The rope is placed between the second and third phalanxes of the fingers who are found to be subjected to vibrating and frictional stresses due to contact with the rope. The back of the arrow should be hooked to the string between the third finger and the second finger, while the fifth finger and the first remain detached and less prone to mechanical stress. The left hand is the hand of the bow or hand on the riser (**a**) and must be positioned so that the knuckles come to draw an angle of about 45 degrees on the vertical to press the grip against the thenar eminence, why it is the eminence that is the most affected by the pressure rather than the fingers of the hand.

Archery is also a very versatile sport, differing in various categories, even if the Olympic bow is the only type of bow admitted to the Olympics. In archery it is essential to have a correct handle, specifically using the right hand to attach the arrow to the rope and to shoot it, while the left hand supports the bow in the shooting position (Figure 1).

Although commonly described as a predominantly mental sport, in which the success of a competition is strongly influenced by anxiety, tension, stress, and pressure of the athlete, archery is also an isometric sport that requires strength, endurance, and precision in movements for a perfect execution of the shot. Furthermore, archery is a sport of strong resistance of the upper body due to the constant use of the arms to which the weight of the shoulder strap is added, thus strongly developing the arm muscles.

Acute damages are caused by performance errors and produce hematomas mostly due to the fracture of bow, arrow, or string during the shot. Frequent hematomas are caused by the absence of protection to the arms and by the return of the rope back in archers who do not comply with the protection measures. Archers may also present palmar petechiae due to friction or trauma [6]. The most observed chronic overuse injuries are found in the shoulders, especially the one holding the arch, and in the arms, and are represented by damage to tendons, ligaments and joints [7,8,9,10,11]. Localized injuries to fingers and hands from overuse are rare; however, the repetitive movements and vibrations produced by the bow handle can also cause damage [12].

Although there is no data on vascular changes in archers in the literature, it is conceivable that the repetitive use of the fingers, involved in the action of maintaining the bow and to tense the rope, the microtraumas and the vibrations associated with shooting can cause over time the development of damage at the level of the capillaries of the fingers, predisposing those who practice archery to the development of Raynaud syndrome and related microvascular abnormalities [13]. Several other causes exist that can induce microvascular disturbances, such as connective tissue diseases (e.g., systemic sclerosis), some chemicals and drugs, anorexia nervosa, extrinsic vascular obstruction, vibration exposure (hand-arm vibration syndrome), traumatic injuries, and hyperviscosity states (paraproteinemia) [14,15,16,17]. Microvessel networks have very complex structures and its alteration causes heterogeneity of blood flow, alterations in oxygen extraction, the appearance of hypoxic areas and the imbalance of hydrostatic and oncotic pressure that leads to edema [18,19,20]. Different physical forces to which people are constantly exposed for work or hobbies can lead to microvascular damage, in fact, it has been shown that guitar players and some workers, such as those who are subjected to radiation (interventional radiologists, cardiologists, surgeons, etc.) or those who work with vibratile devices (miners, grinders, etc.) were more likely to develop damage [15,21,22]. In our case report sport is the basis of the microvascular damage as it is for volleyball players, although surely the type of sport and the frequency of training turn out to be predisposing factors, leading to the hypothesis of a professional pathology. On the other hand it should be pointed out that a physically active lifestyle increases vasodilation and capillary density and exercise is of critical importance for adequate perfusion and metabolic health muscle, on contrary, sedentary lifestyle, obesity, and ageing lead to impairments in the vasodilator response and is associated with capillaroscopic alterations. The increases in skeletal muscle capillary density also contribute to exercise-induced improvements in glucose metabolism independently of other factors, suggesting that increase in capillary density has the potential to mitigate and possibly prevent declines in glucose metabolism in susceptible older adults and potentially reduce progression to impaired glucose tolerance and type 2 diabetes. Seems important to establish the ambivalent nature of sporting activity, assuming that physical activities in which there are frequent traumatisms, exposures to mechanical forces and competitive levels may be more predisposing to microvascular damage, whereas on the contrary an aerobic sport activity could, on the other hand, give benefits both in terms of increased capillary density and reduction of the risks associated with metabolic syndrome [23,24,25].

## 2. Case Report

We refer to a 16-year-old woman, archer, assessed for edema of the right hand and pain in both hands, persisting for about two years and worsening in the last year, especially after the races and prolonged training.

She had no history of autoimmune, cardiovascular, neurological, metabolic, respiratory, or rheumatological diseases, thyroid dysfunction, diabetes mellitus, carpal tunnel syndrome, or primitive Raynaud’s phenomenon. Moreover, she was not smoker neither assumed drugs, alcohol or medical therapies, and she was right-hander.

She had been practicing archery for 5 years, since she was 11, on average 3 h a day for three days a week, plus a competition a week, lasting 1 h 30 min. The arch she used is an arch compound weighed 48 lb (21 kg) and she held it with her left hand. In each training she shot about 100–200 arrows.

On physical examination she had normal vital signs and no significant manifestation of musculoskeletal, neurological, or cardiovascular pathologies were evident. A careful objective examination was conducted without evidence of pathological manifestations. Laboratory tests were performed including, complete blood examination, erythrocyte sedimentation rate, C-reactive protein, anti-nuclear antibody, estractable nuclear antigens antibody, anti-cardiolipin antibody, anti-phospholipids antibody, rheumatoid factor, anti-streptolysin O titer, anti-neutrophil cytoplasmic antibodies, and anti-citrullinated protein antibody, without finding alterations. A nuclear magnetic resonance imaging of the hands and an upper arms ultrasonography doppler study was performed, but no pathological changes emerged (p.e.: thrombosis, vascular dissection or congenital malformations, and alterations of the musculoskeletal apparatus). In particular, she did not present symptoms suggestive of connective tissue diseases (no digital ulcers or skin manifestations suggesting underlying systemic lupus erythematosus or scleroderma). However, since she reported practicing archery at a competitive level, we performed the videocapillaroscopy (NVC) to evaluate any possible microcirculatory change [16].

The NVC study was carried out by using a ×200 optical probe-equipped videocapillaroscope connected to an image analyzer software (VideoCap software 3.0; DS Medica, Milan, Italy). Before the examination, patient was acclimatized at a temperature of 23 °C for 15 min; then a drop of immersion oil was applied to the nailfolds of the second, third, fourth and fifth fingers of both hands in order to increase skin transparency, and all of them were examined [17].

Using the NVC we evaluated capillary density, diameter, and morphology, as well as the vascular array organization and the presence of any microhemorrhages [26], as shown in Table 1.

**Figure 2:**
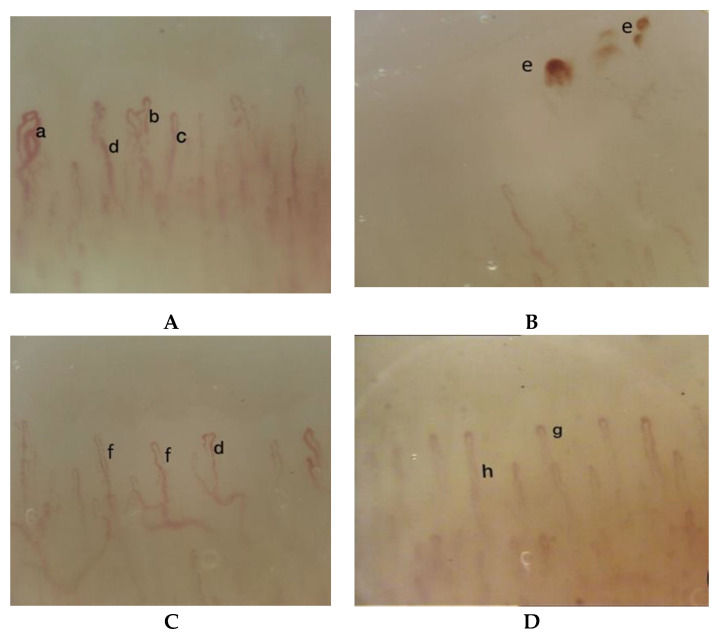
Nailfold videocapillaroscopic images in an archer showing a “non-specific pattern” in right hand (**A**–**C**) and a normal pattern in left hand (**D**): (**A**) Right hand, second finger: (a) ectatic “treble clef” loops capillary, (b) “antler” loops, (c) single tortuosity of the capillary, (d) ectasia of the efferent tract of the loops; (**B**) Right hand, fourth finger: (e) microbleeding; (**C**) Right hand, third finger: (f) granular flow, (d) ectasia of the efferent tract of the loops; (**D**) Left hand, second finger: (g) ”U-shaped” loops, capillaries regularly arranged in a parallel fashion, (h) ratio efferent limb < 2:1.

We found a “non-specific abnormal NVC pattern”, e.g., without findings suggestive of scleroderma, characterized by ectatic “treble clef” capillaries and antler shaped loops, enlarged efferent tracts and single loop tortuosity of the capillaries, granular flow and microhemorrhages (Figure 2). These abnormalities were detected only on the second, third, and fourth fingers of the right hand, while the capillaroscopy findings of the left side were normal. The patient was advised, where possible, to change the posture and the intense tension to which the hands are subjected during the shooting, to perform hand relaxation exercises, to do not take vasoactive substances, to avoid exposing the hands to the cold, pay attention to the appearance of any new symptom and to undergo NVC annually.

The study was conducted after receiving the patient’s informed consent to participate to such clinical investigation and to publish this report, in compliance with the established rules of the Internal Review Board of the University of L’Aquila, Italy, (ex “Comitato etico di Ateneo” D.R. n. 206/2013 modified D.R. n. 46/2017) and conducted in accordance with the 1975 Helsinki Declaration and its subsequent amendments. The patient provided her written informed consent for the publication of this report.

## 3. Discussion

To our knowledge, this is the first study documenting by NVC microvascular anomalies in an archer. The combination of contraction-relaxation is fundamental in archery. Moreover, it has to keep in mind the behavioral asymmetry of the upper limbs: the left upper limb (in the right-handed archer) works in stabilization with the shoulder fixator muscles and the limb abductors, the right arm works, instead, by means of an isotonic contraction performing constant traction of the rope [27]. Therefore, starting from the evaluation of the muscular work performed during the execution of a shot, we can say that the most stressed joints are in the first instance the two scapulo-humeral joints, even if the action takes place thanks to the muscles related to the scapula and the spine [28,29,30].

The most frequent pathologies of archers are those of functional overload, especially in the shoulder corresponding to the hand of the rope, with coraco-acromio-humeral friction syndromes [31]. Due to the repetition of mechanical stress, inflammatory processes can also occur at the level of the elbow (epicondylitis) both at the level of the arm that holds the arch and at the level of the arm that pulls the rope, as at the flexor tendons of the fingers that pull the rope, mainly caused by an abnormal grip of the rope by the fingers that pull it, with loss of tension in one finger and consequent overload of the other [32]. Even the calluses that form on the fingers that hold the rope tight, can unfortunately cause painful symptoms since the strong compression exerted by the callus damages the subcutaneous tissue, such as tendons, nerves, and capillaries that pass here.

Extremely important in archery is the relationship between activation, attention, and muscle tension, in particular, as the activation level increases, the level of muscle activation also increases. In the archers, we can also find the hypothenar or thenar hammer syndrome usually observed in the aneurysmal degeneration, thrombosis, and distal occlusion of the proximal posterior circumflex humeral artery, due to the hammer position held by the hand that holds the arch.

Extremity pain, edema, cold, blue, and pale fingers are signs associated with vascular disorders that require careful evaluation in affected patients. Our young patient started to shoot with the bow at the age of 11 and at 14 presented the first disorders. Changes in endothelial function and reactivity of arteriolar smooth muscle cells have been linked to juvenile growth but it is still unclear to which extent has an impact on microvasculature function [33]. Microvascular reactivity in children and adolescent is related to age and affected by gender during adolescence [33] and is higher in young healthy women respect to men [34]. Vibration increases microcirculation of blood in the skin [35]. Multifactorial pathophysiological mechanism are hypothesized to underlie the vibration induced microcirculatory abnormalities, with interrelations between structural and functional changes of the vasculature wall and the endothelium, disorders of the neural control of the vascular tone and endothelial injury and/or the impairment of the blood flow. However in our girl, the presence of capillaroscopic anomalies exclusively on the right hand, which is the one most subjected to muscle tension and vibrations, makes us suppose that in the latter we can identify the causes of the microvascular changes. In the reported case, the presence of non-specific symptoms led us to document alterations in the microcirculation; however, especially in the early stages, vascular damage can be asymptomatic and hence the need to perform an NVC at least in elite athletes, who have been practicing this sport for several years and with a certain assiduity. An undiagnosed vascular disease for a long time could have devastating consequences. It is, therefore, important to carry out videocapillaroscopic examinations to assess any damage to the fingers and in particular of the digital capillaries. NVC allows evaluation of the skin microcirculation in a simple and non-invasive way and can diagnose, after excluding the most serious diseases, digital vascular anomalies due to repeated trauma of the fingers. For example, the NVC is a tool widely used to diagnose microvascular abnormalities in the hand-arm vibration syndrome [16,17,18,19,20,21,22,23,24,25,26,27,28,29,30,31,32,33,34,35,36] and allows, according to the morphological pattern, the differential diagnosis with vascular damage secondary to autoimmune and connective tissue diseases, such as scleroderma [37]. Furthermore, thanks to its simplicity and speed of execution and its low cost, capillaroscopy can also be easily performed in the follow-up. Repeated trauma and exposition to vibration can constitute potential risk factors for microcirculation causing an increased tendency to vasospasm in digital capillaries. Archery involves an almost constant isometric contraction of the muscles of the hand, forearm, arm, and shoulder; however the greatest tension is concentrated at the level of the fingers that pull the rope and shoot the arrow. In general, little is known about how harmful tension is, especially in athletes. In addition, kinetics, strength, intensity, duration, frequency of exposure, and several other cofactors regulate this dangerousness and should therefore be taken into account. Furthermore, age, sex, smoking, chronic emotional stress and exposure to radiations may also influence the risk of developing microvascular disturbances [21]. Documentation of vascular anomalies in a woman who practices archery at a competitive level suggests extending the study to a larger number of athletes to assess their frequency. Based on what we reported here, we consider a capillaroscopic study useful in athletes who practice this sport and in particular in women who are more prone to autoimmune diseases whose early diagnosis is essential. The detection of vascular anomalies by NVC requires further investigations to exclude occult or developing connective tissue diseases and follow-up. The detection of objective and documented vascular alterations will help the athlete to achieve a greater awareness of her condition and reassure her, while making her understand the need for a follow-up and, where possible, for a change in some lifestyles and if possible in shooting technique.

## 4. Perspective

Archers, whose hands are exposed to vibration, may suffer from microvascular damage. For this reason, they should be able to access an effective health surveillance program able to detect the first signs of such vascular abnormalities. Taking good care of their hands also contributes to improving their archery performance. Here we report a single clinical case that does not allow us to give certain indications to all athletes who practice this sport. However, as the alterations described are consistent with harmful actions that their hands suffer, we believe it is appropriate to conduct a study to ascertain the spread of these alterations and their possible evolution in archers and on this basis, being able to give the correct indications to prevent, correct, and/or keep them under control.

If the correlation between archery and microvascular damage will be confirmed, the execution of NVC becomes indispensable, at least in elite athletes.

Presently, the NVC allows us to have a valid method for the evaluation and diagnosis of microvascular alterations, which in sportsmen are still dangerously ignored in the majority of cases while their spread in archers should still be defined.

This is of high importance for maintaining a good state of health over time and, possibly, mitigate or eliminate the factors that worsen sports performance.

## Figures and Tables

**Table 1:** Capillaroscopic Parameters normal characteristics and our patients features.

Capillaroscopic Parameter	Normal Characteristics	Our Patient
Skin transparency and visibility	Transparent, capillaries clearly visible	Reduced skin transparency due to the presence of edema on the second, third, and fourth fingers of the right hand
Capillary array and architecture	Palisading loops, uniform, evenly spaced, major axis of capillaries perpendicular to the distal row	Within normal range
Capillary morphology	U-shaped or harpin-like	Non-homogeneous
Capillary distribution	Symmetric, homogeneous	Moderate non-homogeneity
Capillary diameter	The diameter of arterial(or afferent) limb can vary from 6 to 19 µm (average value: 11 ± 3 µm). The diameter of the venous (or efferent) limb is generally greater, 8–20 µm (average value: 12 ± 3 µm)	Ectasia of the efferent tract of loops 35 µm
Ratio efferent limb/afferent limb	Ratio efferent limb/ afferent limb < 2:1	Ratio efferent limb/afferent > 2:1
Capillary density	Normally between 9 and 14 inverted U-shaped capillaries evenly distributed in 1 linear mm	Within normal range
Sub-papillary venular plexus	The visibility of the sub-papillary venular plexus is subject to the level of skin transparency	Not visible
Branched capillaries	Evaluating whether the crossovers were single or multiple	Ectatic treble clef capillaries and antler loops
Capillary blood flow	Normally dynamic, no stasis or thrombosis	Granular flow
Abnormalities	No morphological abnormalities: tortuosity, homogeneous enlarged loops, neoformation of capillaries and microbleeding	Tortuosity, homogeneous enlarged loops, microbleeding
